# Quantitative trait loci for resistance to trichostrongylid infection in Spanish Churra sheep

**DOI:** 10.1186/1297-9686-41-46

**Published:** 2009-10-28

**Authors:** Beatriz Gutiérrez-Gil, Jorge Pérez, Lorena Álvarez, Maria Martínez-Valladares, Luis-Fernando de la Fuente, Yolanda Bayón, Aranzazu Meana, Fermin San Primitivo, Francisco-Antonio Rojo-Vázquez, Juan-José Arranz

**Affiliations:** 1 Departamento de Producción Animal, Facultad de Veterinaria, Universidad de León, 24071, León, Spain; 2 Departamento de Sanidad Animal, Facultad de Veterinaria, Universidad de León, 24071, León, Spain; 3 Instituto de Ganadería de Montaña, Centro Mixto Universidad de León-CSIC Finca Marzanas s/n - CP 24346 - Grulleros, León, Spain; 4 Departamento de Sanidad Animal, Facultad de Veterinaria, Universidad Complutense, 28040 Madrid, Spain

## Abstract

**Background:**

For ruminants reared on grazing systems, gastrointestinal nematode (GIN) parasite infections represent the class of diseases with the greatest impact on animal health and productivity. Among the many possible strategies for controlling GIN infection, the enhancement of host resistance through the selection of resistant animals has been suggested by many authors. Because of the difficulty of routinely collecting phenotypic indicators of parasite resistance, information derived from molecular markers may be used to improve the efficiency of classical genetic breeding.

**Methods:**

A total of 181 microsatellite markers evenly distributed along the 26 sheep autosomes were used in a genome scan analysis performed in a commercial population of Spanish Churra sheep to detect chromosomal regions associated with parasite resistance. Following a daughter design, we analysed 322 ewes distributed in eight half-sib families. The phenotypes studied included two faecal egg counts (*LFEC0 *and *LFEC1*), anti-*Teladorsagia circumcincta *LIV IgA levels (*IgA*) and serum pepsinogen levels (*Peps*).

**Results:**

The regression analysis revealed one QTL at the 5% genome-wise significance level on chromosome 6 for *LFEC1 *within the marker interval *BM4621-CSN3*. This QTL was found to be segregating in three out of the eight families analysed. Four other QTL were identified at the 5% chromosome-wise level on chromosomes 1, 10 and 14. Three of these QTL influenced faecal egg count, and the other one had an effect on *IgA *levels.

**Conclusion:**

This study has successfully identified segregating QTL for parasite resistance traits in a commercial population. For some of the QTL detected, we have identified interesting coincidences with QTL previously reported in sheep, although most of those studies have been focused on young animals. Some of these coincidences might indicate that some common underlying loci affect parasite resistance traits in different sheep breeds. The identification of new QTL may suggest the existence of complex host-parasite relationships that have unique features depending on the host-parasite combination, perhaps due to the different mechanisms underlying resistance in adult sheep (hypersensitivity reactions) and lambs (immunity). The most significant QTL identified on chromosome 6 for *LFEC*_1 _may be the target for future fine-mapping research efforts.

## Background

For ruminants reared on grazing systems, gastrointestinal nematode parasite infections represent the class of diseases with the greatest impact on animal health and productivity [1]. Due to the growing incidence of anthelmintic resistance among most parasite species, there is a need for a sustainable control of gastrointestinal nematode (GIN) parasites. Among the potential strategies, enhancement of host resistance through the selection of resistant animals has been suggested by many researchers. Because of the difficulty of routine collection of phenotypic indicators of parasite resistance, information based on molecular markers can be used to improve the efficiency of classical genetic breeding.

Most studies on the detection of QTL for parasite resistance in sheep have been carried out in sheep populations specialised for meat and/or wool production [2,3], and particularly in young animals [4-7]. However, the variety of sheep breeds and nematode species considered in these studies has resulted in little consensus among the results reported.

In the present study, we carried out a genome scan based on a daughter design in a commercial population of Spanish Churra sheep, an indigenous dairy breed from the region of Castilla y León where the traditional breeding system is based on autochthonous grazing breeds. Even when gastrointestinal parasite infections in Churra sheep are moderate, *Strongylid *nematode parasites are known to cause substantial production losses in the Churra flocks due to subclinical infection and reduction of the general immune response [8]. In addition, the infection of young replacement females turned out to pasture for the first time may lead to clinical signs of disease such as diarrhoea and even death in some cases [8].

Previously, we quantified the proportion of the phenotypic variation of four parasite resistance traits that are under genetic control [9]. The occurrence of heritable variation has been observed for the four parasite traits studied, which suggests that genetic improvement is possible for these traits. However, the low heritability estimates obtained for the studied indicators of parasite resistance (ranging from 0.09 to 0.21), together with the difficulty of routinely collecting these phenotypes, suggests that the use of marker assisted selection might be of special interest for enhancing the response to selection of these traits. Based on this, and taking advantage of the genotypic information generated in a previous genome screening program undertaken in Churra sheep, we performed an initial QTL scan for four parasite traits measured in eight half-sib families of the Selection Nucleus of ANCHE (National Association of Spanish Churra sheep Breeders).

## Methods

### Sampled Animals and Measurements

The experimental design used in the present study is the daughter design described by Soller and Genizi [10]. We analysed a total of 322 ewes belonging to eight half-sib families, with an average family size of 40.25 daughters per sire (range: 19-84). Samples from these animals were collected from seven flocks included in the Selection Nucleus of ANCHE (National Association of Spanish Churra sheep Breeders).

As indicators of parasite resistance following natural infection, we used the phenotypes studied by Gutiérrez-Gil et al. [9] for the estimation of genetic parameters of parasite resistance traits in a larger population of Churra sheep (928 ewes). From the 928 animals sampled for parasite resistance traits, those animals belonging to half-family groups with at least about 20 ewes were selected for the present QTL detection experiment, avoiding the analysis of very small families. Hence a total of 322 animals were included in the genome scan analysis reported here. The methodology and techniques used to determine these phenotypes have been described in detail by Gutiérrez-Gil et al. [9]. Below is a brief description of the four phenotypes analysed, followed by a brief comment on the aspect of parasite resistance to which each trait is related:

(i) *FEC*_0_: Faecal egg count per gram (epg) at day 0 of the experiment, when all sampled animals received anthelmintic treatment. A modified McMaster technique was used to determine faecal egg counts. After the anthelmintic treatment the animals were exposed to natural infection in the fields following the normal management used by Churra sheep breeders. After a period of about 60 days, the following three measures were performed.

(ii) *FEC*_1_: Faecal egg count per gram (epg) at approximately day 60 after beginning the experiment. A modified McMaster technique was used to determine faecal egg counts.

(iii) *IgA*: The IgA (*IgA*) levels against a somatic extract of the fourth stage larvae (LIV) from *Teladorsagia circumcincta *were measured using an ELISA test based on the technique described by Martínez-Valladares et al. [11].

(iv) *Peps: *The concentration of serum pepsinogen, as measured by fluorometric determination in a 96-well microtitre plate using a technique adapted from Edwards et al. [12].

The number of eggs per gram of faeces (epg) is a measure of eggs produced by adult female parasites within the host animal and is thought to be a good indicator of the parasite infection status of the host [13]. In addition, the serum anti-*Teladorsagia circumcincta *LIV level (*IgA*) is an indicator of a specific immune reaction to the fourth stage larvae of *T. circumcinta*, the most important parasite in Churra sheep. The serum pepsinogen level is an indicator of gastric damage associated with the progression of larvae to adult stages [14]. In Churra sheep, the increase in serum pepsinogen has been found to be triggered by the action of the LIV and early non-egg-laying adults [11]. Hence, the traits studied are likely to represent different aspects related to the host-parasite interaction during infection.

Age of the animals was sorted in six different levels according to the lambing number (from 1 to 5 years, and 6 or more than 6), and their physiological status varied among four different states (dairy, pregnant, dry-not pregnant or *peripartum*), as we have previously reported [9].

Basic statistics for the four measured traits are given in Additional File 1. Regarding the faecal egg count, the most prevalent genera encountered was *Teladorsagia *(65.5%), followed by *Trichostrongylus *spp. (30.5%), *Nematodirus *spp. (3.1%) and some less frequent genera (1% *Chabertia *spp. and *Oesophagostomum *spp.).

Prior to further analysis, the distribution values for *FEC*_0 _and *FEC*_1_, which were positively skewed, were transformed using a logarithmic transformation [*LFEC*_0 _= ln (*FEC*_0_+1); *LFEC*_1 _= ln (*FEC*_1_+1)]. *IgA *and *Peps *did not require any transformation. The influence of fixed factors and the estimation of genetic parameters for the studied traits have been reported elsewhere [9].

### Data Analysis

#### Genotyping and Linkage maps

A total of 322 ewes from the complete set of animals sampled for parasite resistance traits (928 ewes) were included in a genome scan analysis carried out in Churra sheep to detect QTL for dairy traits [15-17]. Taking advantage of the genotypic information generated in that genome scan and the linkage maps built for the Churra sheep population, we performed a QTL analysis for the four traits related to parasite resistance considered in this experiment. In this genome scan a total of 182 markers (181 microsatellites and 1 SNP) distributed along the 26 ovine autosomes were genotyped across 1,421 animals belonging to 11 half-sib families. The procedures used for the genotyping of the 182 markers have been described in detail elsewhere [16,17]. The linkage map used in the current work was that generated for the most complete Churra sheep population genotyped (1.421 ewes), which has been reported by Gutiérrez-Gil et al. [18]. This map, which was built with the CRI-MAP 2.4 software [19], showed an average marker interval of 17.86 cM [18] and an information content (IC) for QTL detection of about 0.6 [17]. The use of this map for the parasite resistance genome scan allowed a more accurate estimation of the phase of the paternal sires, yielding therefore more reliable QTL results.

#### QTL Analysis

Mapping of quantitative trait loci was performed by the multimarker regression method described by Knott et al. [20] for half-sib designs implemented with the HSQM software [21]. Response variables used in the QTL analysis were the Yield Deviations (YD) [22], which are the records expressed as deviations from the population mean and corrected for the corresponding environmental effects. For each trait, the effects included in the YD calculation were those considered in the estimation of the genetic parameters, which had been shown to have a significant influence on the trait (Flock-Year-Season (5 levels), Lambing Number or age (6 levels), and Permanent Environmental effects for the four traits; the sampling interval was also considered for *LFEC*_1_). When evidence for a significant effect was found in the across-family analysis, the position with the greatest F-value was considered as the most likely location of the QTL, and the within-family analysis was examined to identify the segregating families and to estimate the QTL size effect.

Chromosome-wise significance thresholds were obtained for each trait-chromosome combination by performing 10,000 random permutations of the phenotypic data [23]. QTL effects were considered significant if they exceeded the 5% chromosome-wise significance threshold (p_c_-value < 0.05). Genome-wide p-values were obtained by applying the following Bonferroni correction: *P*_*genomewise*_* = 1-(1-P*_*chromosomewise*_*)*^(1/*r*)^, where *r *indicates the contribution of the chromosome to the total genome length [24]. The *r *parameter was calculated based on the last update of the Australian sheep linkage map [25] (consulted September 2008). The results of the within-family analyses were used to identify the families segregating for each of the QTL identified at the whole population level (those with a within-family p_c _< 0.05, as determined through permutation testing). Correction for multiple traits was not performed due to the preliminary nature of the genome scan so that we could compare our results with other studies [24]. Empirical 95% confidence intervals (95% CI) were calculated by the bootstrapping method [26].

## Results

The regression analysis revealed five significant QTL at the 5% chromosome-wise level on chromosomes 1, 6, 10 and 14, and the QTL on chromosome 6 exceeded the 5% genome-wise significance level. Details regarding the QTL position, significance level and 95% CI calculated for all the QTL identified by the across-family regression analysis are given in Table 1, along with the position and estimated effect for each of the segregating families identified in the within-family analysis.

**Figure 1:**
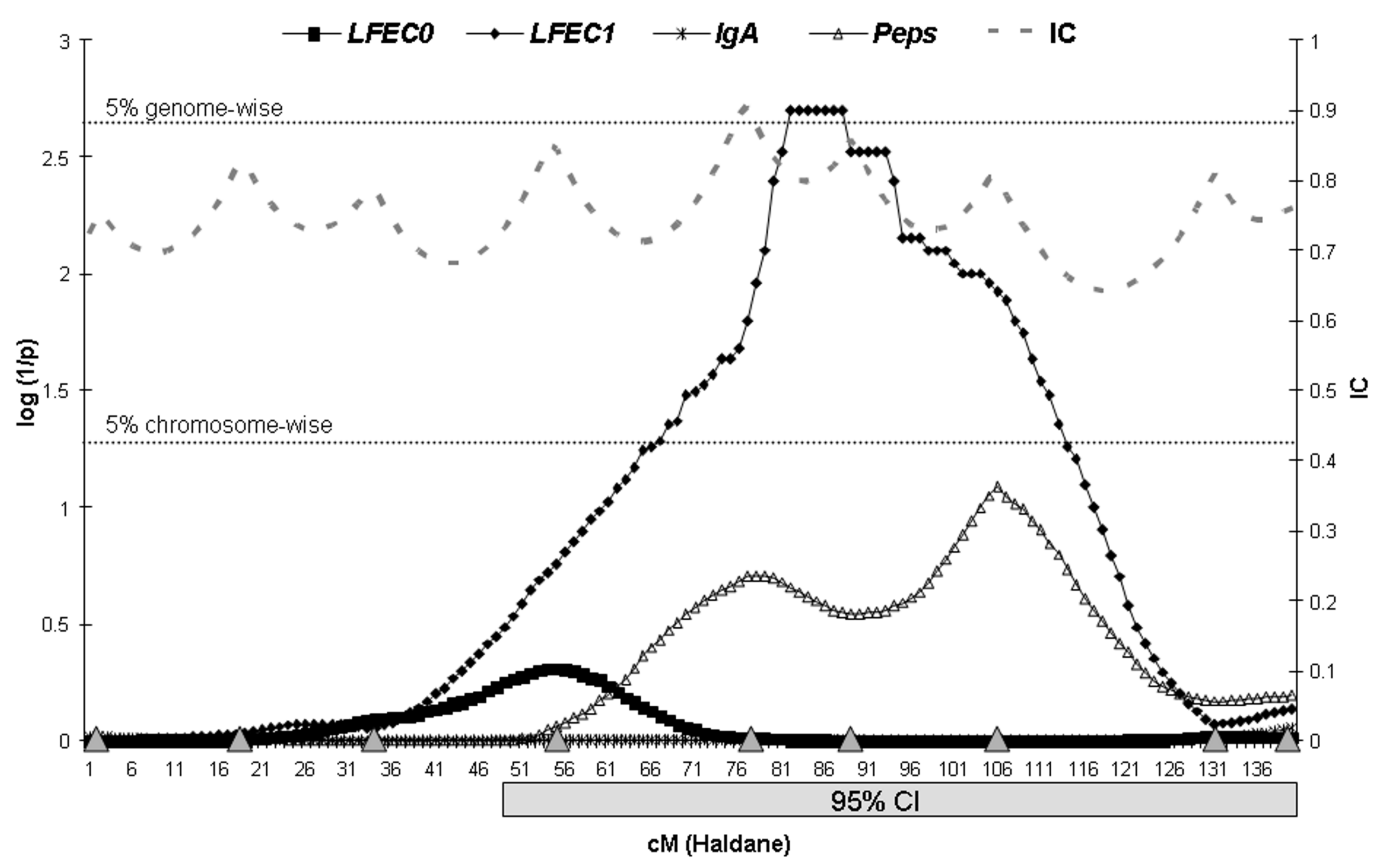
**Across-family statistical profiles obtained on chromosome 6 for the four parasite resistance traits analysed in the present study**. The *x*-axis indicates the relative position on the linkage map (cM Haldane); the y-axis represents the log (1/p_g_-value); the horizontal lines indicate the 5% genome-wise and 5% chromosome-wise significance thresholds. Information content (IC) obtained along the linkage map is represented at the right, on the *y*-axis; beginning at the centromeric end, the triangles on the *x*-axis indicate the relative positions of the markers analysed on this chromosome, which were *INRA133, MCM53, MCMA14, BM143, BM4621, CSN3, CSRD2158, MCM214 *and *BL1038*; confidence interval (95% CI), calculated by bootstrapping analysis of the *LFEC*_1 _QTL, is shown as a grey box at the bottom of the figure.

**Figure 2:**
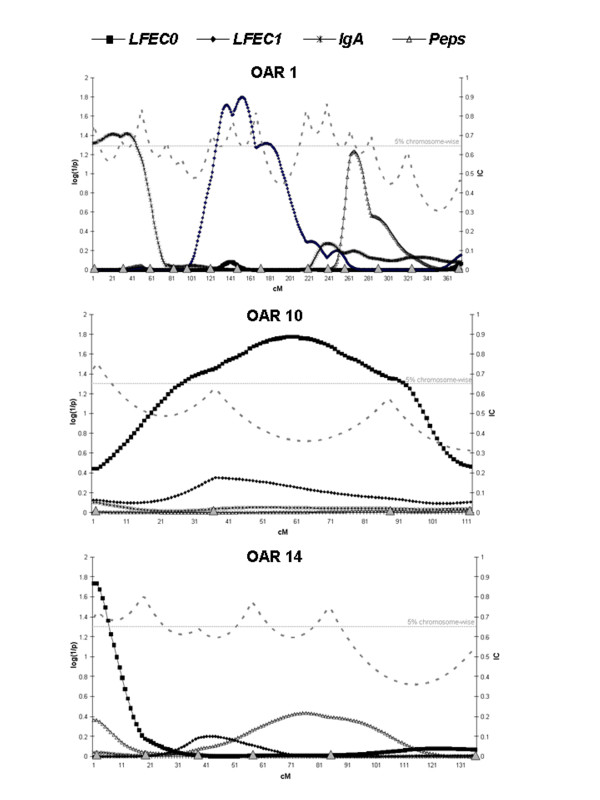
**Across-family statistical profiles obtained on chromosomes 1, 10 and 14 for the four parasite resistance traits analysed in the present study**. The *x*-axis indicates the relative position on the linkage map (cM Haldane); the y-axis represents the log (1/p_g_-value); information content (IC) obtained along the linkage map of each chromosome is represented at the right, on the *y*-axis; the horizontal lines indicate the 5% chromosome-wise significance threshold; beginning at the centromeric end, the triangles on the *x*-axis indicate the relative positions of the markers analysed on each chromosome; see Gutiérrez-Gil et al. [18] for details about marker names and genetic distances.

Significant QTL were found for three out of the four traits investigated. Four of the significant linkage associations identified influenced the faecal egg count, and one chromosomal region was associated with the *IgA *serum indicator. No QTL were observed for *Peps*. The statistical profiles for the four parasite resistance traits obtained along the four chromosomes where the significant QTL were detected are represented in Figures 1 and 2.

The most significant QTL was located on the second half of chromosome 6, within the marker interval *BM4621-CSN3*, and influenced *LFEC*_1 _(Figure 1). This QTL reached genome-wise significance (p_g _= 0.041) and was found to segregate in three out of the eight analysed half-sib groups (Families 1, 2 and 7). For Family 1, which showed the highest significance level, the QTL position suggested by the within-family analysis was coincident with the results of the across-family analysis. For the two other segregating families, the QTL were localised within the first and second downstream marker intervals with regard to the QTL across-family position. Here, it should be noted that the estimation of the across-family QTL position may be biased towards the marker with the highest informativeness in the region, microsatellite marker *BM4621*, for which all the sires included in the study were heterozygous. This discrepancy regarding the within-family QTL positions may explain the large 95% CI obtained for this QTL, which spanned 91 cM of the chromosome length. However, the possibility that the effect detected at the across-family level can be due to different QTL segregating in the different families can not be ruled out. The magnitude of the allelic substitution effect for this QTL in the segregating families ranged from 0.83 (Family 2) to 1.63 (Family 1) phenotypic SD units (Table 1).

**Table 1:** Characterisation of QTL influencing parasite resistance traits that exceed the 5% chromosome-wise significance threshold in the commercial population of Spanish Churra sheep analysed in this study

ACROSS-FAMILY ANALYSIS				WITHIIN-FAMILY ANALYSIS		
	
Chr.^1^	Trait **Position^2^** [95% CI]^3^	Flanking interval^4^	P_c_^5 ^(*P*_*g*_)^6^	Segregating families P_c_^7^	Position^8 ^Flanking interval^9^	Size effect^10 ^(SD units)
1	*IgA * 35 cM [1-320].	BMS835-ILSTS044	0.038	Family 1 0.005	38 cM BMS835-ILSTS044	0.111 (1.70 SD)
	*LFEC*_1 _ 152 cM [122-374]	INRA006-BMS574	0.016	Family 8 0.013	134 cM INRA006-BMS574	0.129 (1.31 SD)

6	*LFEC*_1 _ 84 cM [49-140]	BM4621-CSN3	0.002 (*0.041*)	Family 1 0.002 Family 2 0.041 Family 7 0.049	79 cM BM4621-CSN3 113 cM CSRD2158-MCM214 105 cM CSN3-CSRD2158	0.160 (1.63 SD) 0.082 (0.83 SD) 0.117 (1.19 SD)

10	*LFEC*_0_ 59-60 cM [1-95]	BM4621-CSN3	0.018	Family 7 0.014	65 cM BMS975-TGLA441	0.324 (2.53 SD)

14	*LFEC*_0_ 1-2 cM [1-125]	TGLA357-CSRD247	0.018	Family 1 0.029 Family 6 0.015	2 cM TGLA357-CSRD247 1-2 cM TGLA357-CSRD247	0.137 (1.07 SD) 0.136 (1.06 SD)

^1 ^Chromosome number

^2,8 ^Position (cM Haldane) of the chromosome where the maximum F-statistic value was obtained in the across- and within-family analyses, respectively

^3 ^The 95% confidence interval obtained by bootstrapping analysis [26] is shown in square brackets (cM Haldane)

^4,9 ^Markers flanking the position of the maximum F-statistic in the across-family and within- analysis, respectively. Markers in bold caps are < 1 cM from the maximum F-statistic

^5,7 ^P_c _= chromosome-wide p-value obtained by permutation test for that position [23]

^6 ^P_*g *_= genome-wide p-value for that position obtained by applying the following Bonferroni correction: *P*_*genomewide*_* = 1-(1-P*_*chromosomewise*_*)*^(1/*r*)^, where *r *indicates the contribution of the chromosome to the total genome length [24]; only indicated for *P*_*g *_< 0.05

^10 ^Magnitude of the allelic substitution effect calculated for each segregating family, expressed in units of the trait (egg count/g faeces for *LFEC*_0 _and *LFEC*_1_, D.O ratio for *IgA *and mUTyr *Peps*) and in phenotypic SD units of the analysed YDs (value in brackets)

On chromosome 1, a QTL associated with *LFEC*_1 _was also found at the 5% chromosome-wise significance level. This QTL was found in the central region of the chromosome (152 cM). Close to the proximal end of the same chromosome, there was evidence for an additional 5% chromosome-wise significant QTL influencing *IgA*. The other two significant QTL identified by the across-family analysis were found on chromosomes 10 and 14 and showed effects on *LFEC*_0_. The QTL on chromosome 10 was located approximately in the middle of the chromosome, whereas the QTL on chromosome 14 was found at the proximal end, close to the first marker analysed on this chromosome.

For each of the QTL identified at the 5% chromosome-wise level, only one family within the population was found to be segregating. The exception to this was the QTL identified on chromosome 14, where the within-family analysis indicates that two of the eight sires are likely to be heterozygous for this QTL (Families 1 and 6). The QTL position suggested by the within-family analysis for these two families was coincident with that estimated in the across-family analysis. The magnitude of the estimated allelic substitution effects for the QTL identified at the 5% chromosome-wise level ranged from 0.83 (chromosome 6 QTL for *LFEC*_1_, Family 2) to 2.53 (chromosome 10 QTL for *LFEC*_0_, Family 7) phenotypic SD units.

## Discussion

Via a genome scan analysis, this study, based on the daughter design described by Soller and Genizi [10], has identified five QTL influencing parasite resistance traits on four sheep autosomes. Considering that two independent traits were analysed (according to a principal component analysis performed with the SAS^® ^package [27]; results not shown), the numbers of tests in our experiment expected by chance alone to be significant at the 5% genome-wise and chromosome-wise level are 0.13 and 2.6, respectively. We identified one and four significant associations in our across-family analysis for these respective significance levels, providing evidence in favour of genuine segregating QTL for parasite resistance traits in the studied population of Churra sheep.

By adapting the method proposed by Weller et al. [28] to our experimental conditions (e.g., the number of ewes and families analysed, marker density and marker informativeness), we estimated that the power of this experiment to detect a QTL with two alleles that occur with equal frequency and influence a trait with a heritability of 0.20 varied between 16% (0.3 phenotypic SD units) and 42% (0.5 phenotypic SD units) according to the magnitude of the allelic substitution effect that we considered. This estimation was performed assuming a type I error rate of 0.05 and 10% recombination between a marker and the QTL. Hence, we should take into account the fact that the low number of animals analysed in the regression analysis had an important negative influence on the statistical power of the experiment, and that a substantial proportion of genuine segregating QTL, especially those with small effects, may not have been identified by the across-family regression analysis performed. Therefore, we suggest that some of the other regions that were identified at a lower significance level in the across-family analysis might represent genuine QTL segregating in individual families. Some of these weak associations, e.g., QTL identified at the 10% chromosome-wise significance level for *Peps *on chromosomes 1, 2 and 24, *IgA *on chromosomes 9 and 13, and *LFEC*_1 _on chromosome 26 (data not shown), might be confirmed if additional animals were to be included in the analyses.

The lack of coincidence among the QTL identified for the different traits analysed here supports our previously mentioned hypothesis that the traits studied may represent different aspects of the host-parasite interaction during infection. It is possible that the QTL detected for *IgA *and *Peps *could be related to the early response to incoming larvae (i.e., hypersensitivity reactions), whereas the QTL for faecal egg counts may be associated with the ability to avoid the development of adult parasites. This agrees with the observations reported by Davies et al. [7], who did not find any coincident QTL between parasitic traits and IgA activity. The lack of coincidence between the QTL influencing *LFEC*_0 _and *LFEC*_1_, although intriguing, agrees with certain differences observed regarding the correlations between these two traits and the serum indicator traits [9]. As suggested in that work, this could be related to the limited sample period between the faecal egg counts, which could indicate that *LFEC*_1 _is a better indicator of the initial immune response triggered by larvae at the beginning of infection.

On the other hand, the allelic substitution effects estimated for the QTL reported herein are likely to be overestimated as a result of the low power of the experiment at the sire-marker level. As shown by Lynch and Walsh [29], the lower the power, the more the effects of a detected QTL are overestimated. Hence, the genuine QTL effects are likely to be much smaller. This result would be in accordance with the work of Houle et al. [30], who suggested that parasite resistance is likely to be controlled by several loci and, therefore, may receive a strong mutation input, which generates genetic variation. This agrees with the complexity of the physiological processes that lead to nematode resistance [31].

In order to compare our QTL analysis results with chromosomal regions previously identified in sheep in relation to parasite resistance traits, we consulted the Sheep Quantitative Trait Loci (QTL) database [32] and other reports available in the literature. We found that some previously published QTL are coincident with the results reported herein. It is worth noting, however, that most of the QTL mapping studies targeting parasite resistance traits in sheep have typically used experimentally challenged animals, and that the parasite species considered vary between studies. In addition, most of the previously reported studies consider parasite resistance traits measured in young animals, mainly meat production lambs.

Marshall et al. [33] recently reported a QTL on chromosome 1 for *Haemonchus contortus *faecal egg count in 13-month-old Australian sheep. This QTL is close to the marker *ADMST4*, which maps within the flanking interval of the chromosome 1 QTL reported here for *LFEC*_1_. At the proximal end of the same chromosome, within the marker interval *EPCDV010-ILSTS044*, Díez-Tascón et al. [5] reported a within-family QTL for faecal strongyle egg count and an across-family significant QTL for adult *T. columbriformis *recovered from the gastric contents of outcrossed lambs at slaughter. These significant associations co-localise with the position of the chromosome 1 QTL influencing *IgA *that was identified in Churra sheep in our analysis.

On chromosome 6, Beh et al. [4] reported a genome-wise significant QTL for faecal *T. columbriformis *egg count in lambs after primary challenge. This QTL was confirmed to have a chromosome-wise significance following a secondary challenge and mapped to the interval between markers *MCMA22 *and *MCM214*. According to the latest version of the Australian Sheep Linkage Map (v 4.7) [25], the first of these two markers is 16 cM distal to *CSN3 *(male map), one of the markers flanking the genome-wise significant QTL identified by our across-family regression analysis.

On chromosome 14, Davies et al. [7] reported three QTL related to *Nematodirus *egg count in Scottish blackface lambs that were located in the last third of the chromosome, whereas the QTL for *LFEC*_0 _that we identified mapped to the centromeric end of chromosome 14.

Considering the low resolution of the preliminary genome scans that have been reported thus far regarding QTL position, some of these coincidences might indicate common underlying loci affecting parasite resistance traits. However, this possibility should be confirmed with further studies. Taking into account the high degree of variation between different experiments due to factors such as the type of parasite exposure (natural or artificial challenge), the parasite species, the phenotypic indicators and the breeds of sheep studied, the identification of non-coincident QTL in different experiments may suggest the existence of complex host-parasite relationships that have unique features that depend on the host-parasite combination.

Curiously, our analysis did not find any significant association within two of the regions for which consensus has been found in different studies. These are the regions close to *IFNG *on chromosome 3 [7,34] and the histocompatibility complex (MHC) region on chromosome 20 [7,35,36]. This discrepancy may be explained by the fact that the studies that found significant associations in these two regions were focused on lambs, whereas our study considered adult ewes. Marshall et al. [33] reported an important age and/or immune status specificity of the QTL for resistance to *Haemonchus contortus *that they identified in Australian sheep. This specificity is based on the low overlapping levels observed for the QTL that influenced the faecal egg counts measured in animals 6 and 13 months of age. This kind of age-specific mode of action could apply to most parasite infections, which would provide support at the genetic level for the hypothesis suggested by Stear et al. [37] that describes the different mechanisms controlling GIN parasite infections in lambs (antibody response) and adult sheep (hypersensitivity reaction). Also, Balic et al. [38] suggested that the genes that control key mechanisms preventing the establishment of worms in primary infections are different from those involved in subsequent infections. This idea is based on the different pathways that are involved in innate and acquired resistance. However, this hypothesis is challenged by the fact that overall immunity has been successfully achieved through selection for acquired resistance rather than via resistance to primary exposure to worms [31]. All these observations highlight the complexity of parasite resistance and the difficulty of completely understanding the genetic architecture of the physiological mechanisms underlying resistance as well as resilience. As mentioned by Dominik [31], consistency in protocols, experimental materials and analysis approaches would facilitate the generation of phenotypic information that would help to increase our knowledge on this topic.

## Conclusion

In conclusion, we present evidence for a significant number of QTL that influence parasite resistance indicator traits in adult dairy sheep. Some of these linkage associations appear to confirm and support the presence of previously published QTL for parasite resistance in lambs, which could indicate that common genes underlie these traits throughout an animal's life. This study represents a starting point for a better understanding of the genetic architecture of parasite resistance in Churra dairy sheep. Further fine-mapping research efforts focused on the most promising regions, e.g., the genome-wise significant QTL identified on chromosome 6, might be simplified as sheep SNP chips become affordable.

## Competing interests

The authors declare that they have no competing interests.

## Authors' contributions

BG-G coordinated the genotyping experiments, performed error-checking on genotype data, contributed to interpretation of results and drafted the manuscript. JP, AM and MMV obtained the parasite resistance phenotypic data by collection and analysis of the corresponding samples. LA and YB performed microsatellite genotyping. LFdlF participated in the design and coordination of the study, performed the analyses of genetic parameters and helped to draft the manuscript. FSP selected the animals to be sampled and compiled genealogical information. FARV supervised the collection of phenotypic data and revised the manuscript. JJA conceived of the study, selected the initial marker panel, performed QTL analyses and participated in drafting the manuscript. All authors read and approved the final manuscript.

## Supplementary Material

Additional file 1 **Descriptive statistics of phenotypes analysed in this study**. The data provided represents basis statistic of parasite resistance traits including: the total number of observations analysed, mean, range, percentage of 0-values and SD for each studied trait. Click here for file
