# Supplementary material for: Quantitative trait loci for resistance to trichostrongylid infection in Spanish Churra sheep
Source: Genet Sel Evol. 2009 Oct 28;41(1):46. doi: 10.1186/1297-9686-41-46 (PMC2776584; doi:10.1186/1297-9686-41-46)
Supplement: Additional file 1 — Descriptive statistics of phenotypes analysed in this study. The data provided represents basis statistic of parasite resistance traits including: the total number of observations analysed, mean, range, percentage of 0-values and SD for each studied trait. [file 1297-9686-41-46-S1.DOC]

***Table 1.***  *Descriptive statistics of phenotypes related to parasite resistance analysed in this study. The total number of observations analysed, mean, range, percentage of 0-values and SD are given for each studied trait. Note: FEC_0_ and FEC_1_ are presented as untransformed values and refer to the total number of eggs (all genera of parasites).*

| **Trait** | **Total number of observations**  ***(number of ewes)*** | **Mean** | **Minimum** | **Maximum** | **% 0-values** | **SD** |
| --- | --- | --- | --- | --- | --- | --- |
| ***FEC_0_***  Eggs/g faeces | 1513  *(928)* | 260.01 | 0 | 6062 | 12.89 | 237.01 |
| ***FEC_1_***  Eggs/g faeces | 1301  *(928)* | 104.11 | 0 | 3327 | 31.82 | 134.50 |
| ***Peps*** (mUTyr) | 1175  *(928)* | 231.92 | 0 | 1583 | 1.96 | 199.53 |
| ***IgA***  (D.O ratio) | 1160  *(928)* | 0.23 | -0.56 | 2.32 | 1.64 | 0.28 |
